# Is the relationship between chronic pain and mortality causal? A propensity score analysis

**DOI:** 10.1097/j.pain.0000000000003336

**Published:** 2024-07-09

**Authors:** Eva Ryan, Hanna Grol-Prokopczyk, Christopher R. Dennison, Anna Zajacova, Zachary Zimmer

**Affiliations:** a Department of Mathematics and Statistics, University of Limerick, Limerick, Ireland; b Department of Sociology, University at Buffalo, State University of New York, New York, NY, United States; c Department of Sociology, University of Western Ontario, London, ON, Canada; d Department of Family Studies and Gerontology and Global Aging and Community Initiative, Mount Saint Vincent University, Halifax, NS, Canada

**Keywords:** Chronic pain, Older adults, Mortality, Propensity score matching, Inverse probability weighting, Survival analysis

## Abstract

Chronic pain is a serious and prevalent condition that can affect many facets of life. However, uncertainty remains regarding the strength of the association between chronic pain and death and whether the association is causal. We investigate the pain–mortality relationship using data from 19,971 participants aged 51+ years in the 1998 wave of the U.S. Health and Retirement Study. Propensity score matching and inverse probability weighting are combined with Cox proportional hazards models to investigate whether exposure to chronic pain (moderate or severe) has a causal effect on mortality over a 20-year follow-up period. Hazard ratios (HRs) with 95% confidence intervals (CIs) are reported. Before adjusting for confounding, we find a strong association between chronic pain and mortality (HR: 1.32, 95% CI: 1.26-1.38). After adjusting for confounding by sociodemographic and health variables using a range of propensity score methods, the estimated increase in mortality hazard caused by pain is more modest (5%-9%) and the results are often also compatible with no causal effect (95% CIs for HRs narrowly contain 1.0). This attenuation highlights the role of confounders of the pain–mortality relationship as potentially modifiable upstream risk factors for mortality. Posing the depressive symptoms variable as a mediator rather than a confounder of the pain–mortality relationship resulted in stronger evidence of a modest causal effect of pain on mortality (eg, HR: 1.08, 95% CI: 1.01-1.15). Future work is required to model exposure–confounder feedback loops and investigate the potentially cumulative causal effect of chronic pain at multiple time points on mortality.

## Abstract 2

Supplemental Digital Content is Available in the Text.

Using propensity score methods, we investigate whether chronic pain causally increases mortality among older American adults. Evidence, albeit equivocal, of a modest effect is found.

## 1. Introduction

Chronic pain (sometimes abbreviated as “pain” herein) is a potentially debilitating condition affecting an estimated 20% of American adults.^20^ Pain is known to impact many facets of life, including quality of life,^34,48^ psychological wellbeing,^4^ work productivity,^12,44^ and social functioning.^24^ Whether chronic pain affects not only the quality of life but also the *quantity* of life, however, is less clear.

Net of spurious associations due to confounding, one might expect pain to influence mortality for a number of reasons. Pain may reduce mobility^52^ and functional ability,^53^ which in turn may have detrimental effects on both physical and mental health,^29,60^ leading to greater mortality risk. Another plausible causal pathway is through the use of opioid analgesics for pain management. Opioids have been shown to increase mortality through multiple mechanisms, especially cardiovascular deaths.^70,87^

Empirical research on chronic pain and mortality is characterized by a number of unresolved issues. One is the degree to which pain and mortality are associated net of confounding variables. There is a high degree of heterogeneity in observational studies of this association,^77^ with some finding a significant positive association after adjusting for potential confounders^50,55,63,75^ and others finding no association.^3,23,51,76,88,104^ This discordance may be attributable to methodological differences, such as different follow-up lengths, population characteristics, methods of analysis, confounder adjustments,^77^ or pain phenotypes.^78^ Other possible explanations may be contextual, such as different healthcare systems across countries.^56,91^

A second unsettled issue—and a more contentious one—is the degree to which any association between pain and mortality is causal. Randomised control trials remain the “gold standard” of causal research, but conducting trials of long-term pain exposure is infeasible and unethical. This necessitates the analysis of observational data. However, robust methods specifically designed for causal analysis of observational data^36^ have rarely been applied in pain/mortality research. Many existing studies do not apply an explicitly causal approach but, as is often the case in observational research, use language that could be interpreted as agnostic to causality or as implying causality.^33^

A handful of studies explicitly address the causal relationship between pain and mortality. An analysis of U.S. National Health and Nutrition Examination Survey data found that pain had a causal effect on 3- and 5-year mortality mediated by opioid prescriptions.^40^ Pain was also found to affect mortality in an analysis of the English Longitudinal Study of Ageing (ELSA).^79^ This prior study adjusted for age, sex, education, and wealth, but treated all other variables (multiple health, lifestyle, social and psychological factors) as mediators.

Whether pain has a causal effect on death or is only associated with death due to confounding factors has important implications for the design of interventions to reduce mortality. The aim of this study is to expand our understanding of the pain–mortality relationship by using propensity score methods to explicitly address the question: “Does pain have a causal effect on mortality among older American adults?”

## 2. Methods

### 2.1. Data

This study uses data from the Health and Retirement Study (HRS).^35^ The HRS is sponsored by the National Institute on Aging (grant number NIA U01AG009740) and is conducted by the University of Michigan. Detailed documentation of the study design and data collection is available on the HRS website.^41^ In brief, the HRS uses a multistage area probability sampling design with geographical stratification and clustering. African-American and Hispanic adults are oversampled. Sampling weights are provided in the HRS datasets to account for differential selection probabilities. The HRS began in 1992 with a cohort of 50- to 60-year-old respondents. The Asset and Health Dynamics Among the Oldest Old study was established 1 year later to study a cohort aged 70 years and older. By 1998, the HRS and Asset and Health Dynamics Among the Oldest Old cohorts were combined along with 2 new birth cohorts to fill the age gaps, making the HRS a nationally representative sample of non-institutionalised adults aged over 50 years in the United States.^80^ Study participants are followed up every 2 years by telephone or in-person interviews.

This study analyses community-dwelling adults aged 51 years and above from the 1998 HRS sample. It follows this sample over a 20-year period from 1998 to the end of 2018, monitoring their mortality over this period. Of the 20,003 participants aged 51+ years at baseline in 1998, 32 were excluded due to missing pain data, yielding an analytic sample of 19,971 participants.

### 2.2. Variables

#### 2.2.1. Exposure

The 1998 wave of the HRS included multiple questions on pain experience. Participants were first asked, “Are you often troubled by pain?” This question is believed to capture persistent rather than transient or trivial pain,^32^ with previous research finding that study participants reported being “often troubled by pain” less than half as often as they reported “any pain in the last 30 days.”^11^ Those who responded yes to the first pain question were then asked, “How bad is the pain most of the time: mild, moderate, or severe?” We defined pain “exposure” as reporting moderate or severe pain at the 1998 wave, with no pain or mild pain as a control. We chose these groupings because prior research has found that moderate and severe pain, but not mild pain, are significant predictors of mortality in older adults.^32,78^ Sensitivity analyses comparing severe pain exposure to none/mild/moderate pain, comparing any pain exposure to no pain, and comparing just severe pain to no pain were conducted and gave similar results to the severe/moderate vs none/mild pain groupings (see Appendix 2 for supplemental results, http://links.lww.com/PAIN/C93).

#### 2.2.2. Outcome

The outcome of interest is survival over 252 months from January 1998 (beginning of the 1998 HRS survey year) to December 2018 (end of the 2018 survey year). HRS mortality data are essentially complete. Mortality information is collected in 2 ways: either from family members when attempting to contact a participant for the next survey wave, or through linkage to the National Death Index.^101^ The month and year of death are reported for deceased participants.

#### 2.2.3. Confounders

Careful consideration of both potential confounders and appropriate methods to adjust for them is key to drawing causal inferences from observational data.^36^ This decision cannot be entirely data-driven and must use expert knowledge, as statistical associations alone generally cannot distinguish between mediators (which should not be adjusted for) and confounders (which should be).^96^ The biopsychosocial model of pain adapted for older adults suggests that chronic pain experience is influenced by a range of factors, including biological (eg, sex, age, body mass index [BMI], smoking status, health conditions), psychological (eg, depression), and social (eg, race, socioeconomic status [SES], social isolation) ones.^57^ Similar biological, psychological, and social factors have also been associated with differential mortality.^26,37,64,100,102^ Thus, the relationship between pain and mortality may be conceptualised as a complex causal framework encompassing demographic characteristics, socioeconomic factors, health behaviours, psychological factors, and medical conditions.^105^ A comprehensive confounder adjustment set spanning each of these categories was chosen a priori for this analysis based on the existing pain and mortality literature, as detailed below.

There is evidence in the literature of demographic disparities in chronic pain,^32,59^ with characteristics including increased age, female sex/gender, being divorced or separated, and geographic location identified as risk factors.^58,75,92,106^ Similar demographic disparities in life expectancies and mortality have also been observed.^37,64,81,100^ Demographic variables included as potential confounders in this analysis were age in years in 1998; sex (male, female); race/ethnicity (non-Hispanic White, non-Hispanic Black, Hispanic, non-Hispanic Other); marital status (married, separated/divorced, widowed, never married, other); household size; number of children; region of residence (Northeast, Midwest, South, West); and urbanicity (urban, suburban, ex-urban/rural).

In addition, it is well established that chronic pain is inversely associated with SES.^58^ Indicators of lower SES such as less wealth and lower educational attainment have also been associated with increased mortality risk.^21,26^ SES variables included as potential confounders in this study were the highest level of education (no degree, high school diploma, 4-year college degree, graduate degree); household wealth quartile; employment status (employed, unemployed, retired, not in labour force); food security (“In the last 2 years, have you always had enough money to buy the food you need?” yes, no); veteran status (yes, no); and health insurance type (uninsured, any private insurance, public insurance only). We also included a variable about the importance of religion (very important, somewhat important, not too important), as the centrality of religion in individuals' lives has been associated with both differential pain experience^22^ and mortality.^39^

A range of chronic conditions including diabetes, cancer, cardiovascular conditions, and depression have been associated with greater pain burden in older adults.^46,65,75,105^ These conditions are also well recognized as causes of death.^17,47,49^ Other key health factors known to be associated with both pain and mortality include BMI^28,67,69,86^ and smoking.^30,58^ Health status variables included as potential confounders in this study were active cancer (yes, no); diabetes (yes, no); chronic lung disease (yes, no); angina (yes, no); stroke (yes, no); heart condition (yes, no); arthritis (yes, no); BMI category (underweight BMI < 18.5, normal weight 18.5 ≤ BMI < 25, overweight 25 ≤ BMI < 30, obese 1 30 ≤ BMI < 35, obese 2 35 ≤ BMI < 40, obese 3 BMI 40+); smoking status (never smoker, former smoker, current smoker); and mental health status as measured by the 8-item version of the Center for Epidemiological Studies-Depression Scale (0-8, with higher scores indicating more depressive symptoms).^42^

We note that the association between chronic pain and depression is well established.^10^ However, the direction of the relationship between these conditions is complex and likely reciprocal,^13,16^ partly due to shared neural mechanisms.^38^ Longitudinal studies have found that depression is an upstream risk factor for pain onset,^14,19^ and we assume this is the case in our main analyses. On the other hand, pain may also increase depression risk,^74^ for example, as a maladaptive response to pain-related disability.^82^ The potentially bidirectional nature of the pain–depression relationship complicates our investigation of the effect of pain on mortality, as it is unclear if depressive symptoms should be included as a cause or as a result of pain in the causal model (ie, as a confounder or a mediator in our analyses). As detailed later in this section, we contend with this ambiguity by repeating our analyses with depressive symptoms treated as a mediator rather than a confounder.

### 2.3. Analysis

**Figure 1.**
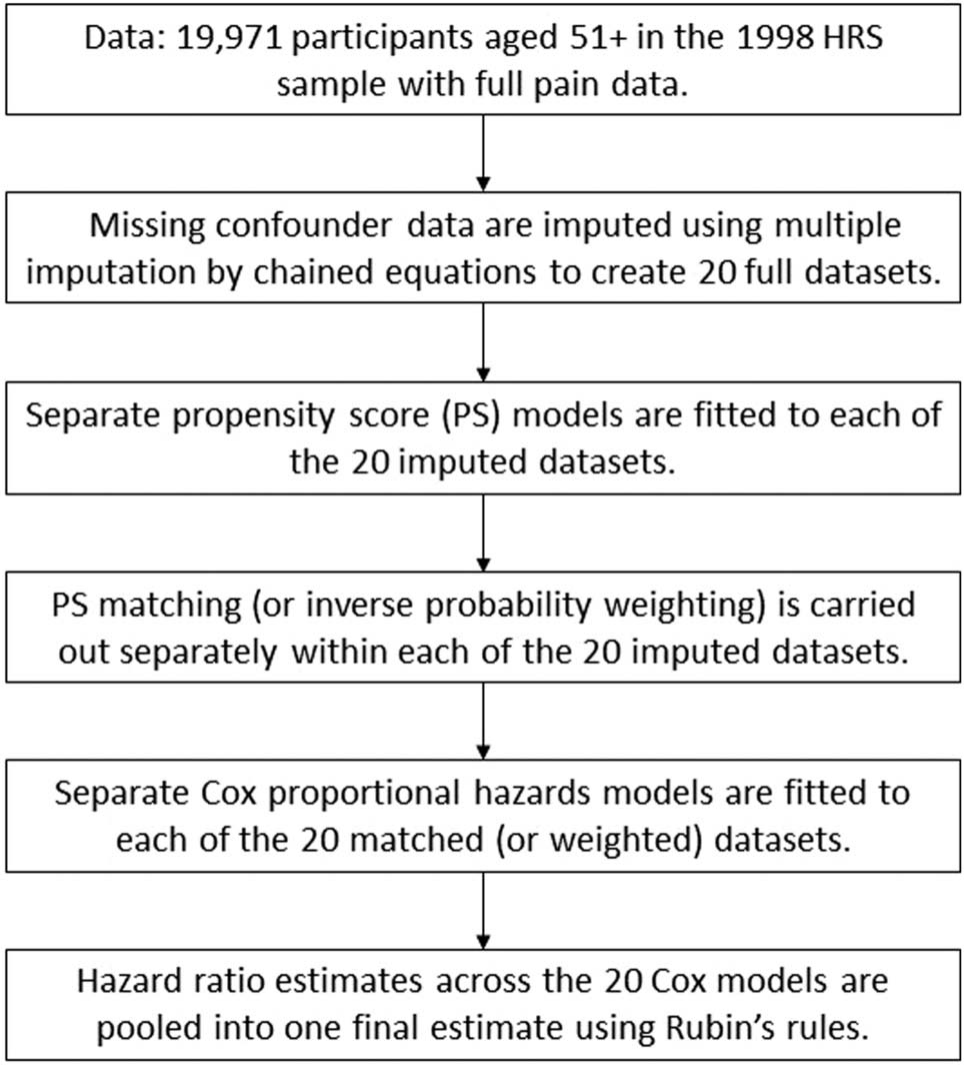
Flowchart summarizing the analytic procedure.

As detailed in the following subsections, we use propensity score approaches and Cox proportional hazard models to analyze the data. Missing data are handled using multiple imputations. An overview of the analytical process is provided in Figure 1.

#### 2.3.1. Missingness and imputation

There was a small amount of missingness on the 1998 variables chosen for inclusion as covariates in the propensity score model. As adult height is quite consistent over time, missing height data needed to calculate the BMI categories were first propagated forward using a last observation carried forward approach from the first HRS wave in 1992. Height values starting from the 2018 wave were then propagated backwards to fill some of the remaining missing height values. After this step, the remaining missing values across all 1998 covariates were imputed using multiple imputations by chained equations.^90^ Variables included in the analyses were used in the imputation models. Propensity score methods and Cox proportional hazard models were applied separately to each of the 20 imputed datasets. The results were then pooled across imputations using Rubin's rules, an approach designed to account for both within-imputation and between-imputation variance.^72^

#### 2.3.2. Propensity score approach

A key assumption for causal interpretation is exchangeability, meaning that the pain-exposed and -unexposed groups have similar distributions of background characteristics and differ only by exposure. This ensures that differences in outcomes between groups can be solely attributed to pain exposure. Exchangeability is achieved in controlled trials by randomization but can also be achieved in observational studies if the conditional probability of exposure depends only on measured covariates (confounders), meaning there is no unmeasured confounding.^36^

Propensity score methods are used to achieve balance on measured confounders across exposed and unexposed groups. The propensity score is the probability of exposure conditional on observed background characteristics. Conditional on the propensity score, the distribution of these background characteristics will be similar in the exposed and unexposed groups.^8^ For our propensity score model, we fit a multiple logistic regression model with pain exposure as the dependent variable and demographic, socioeconomic, and health variables as predictor variables. This model was then used to predict the probability of pain exposure for each participant, and these probabilities were used as propensity scores.

After calculating the propensity scores, we used 2 different methods to achieve balance across the exposed and unexposed groups: propensity score matching and inverse probability weighting (IPW). Propensity score matching involves creating a matched sample by pairing pain-exposed participants with unexposed participants based on the similarity of their scores. Our primary matching approach, often found to outperform other approaches (see Appendix 1, http://links.lww.com/PAIN/C93), was one-to-one nearest neighbour caliper matching without replacement. Inverse probability weighting involves weighting the sample such that the pain-exposed and -unexposed groups have similar distributions of background characteristics. We also used doubly robust models and regression-adjusted matching, which account for confounding both in the propensity score model and in the survival model for the outcome. Finally, as there are many different approaches to propensity score matching, we conducted additional analyses using alternative matching approaches. These were 1-to-1 matching with replacement, 2-to-1 matching with replacement and without replacement, and optimal full matching. Details of all propensity score approaches are included in Appendix 1 (http://links.lww.com/PAIN/C93).

After applying propensity score methods, it is important to perform balance diagnostics to assess whether sufficient balance on background characteristics between the exposed and unexposed groups has been achieved.^6^ Balance is typically assessed by comparing the difference in means and proportions for the various background variables between the exposed and unexposed groups. It is advisable to calculate standardized differences, as they allow variables of different scales to be compared and they are not influenced by sample size.^1,5^ As a general rule, absolute standardized differences below 0.10 are considered indicative of good balance.^7,8^ For this study, the mean and range of standardized absolute mean differences across imputations are summarized in a covariate balance plot, with a dashed vertical line marking the 0.10 absolute standardized mean difference (ASMD) cut-off.

The goal of propensity score methods is to adjust for all possible confounders such that the causal effect of the exposure on the outcome can be identified. When selecting the confounder adjustment set to include in the propensity score model, it is important to avoid adjusting for intermediate variables on the causal pathways between exposure and outcome (mediators of the causal effect). Such “over-adjustment” can introduce serious bias.^93,95^ So-called “collider” selection bias could also be introduced by adjusting for variables that are not confounders, depending on their position in the underlying causal structure.^54^ Causal directed acyclic graphs (DAGs) are useful tools to visualize the exposure and outcome of interest along with all related variables and the proposed causal pathways between them, aiding the appropriate identification of confounders.^84^ We include a DAG to provide such a visualization.

For some variables, determining their likely position in the causal framework was difficult. Specifically, as discussed in more detail in the Confounders section, pain and depression are believed to have a reciprocal relationship^13^ wherein pain may also cause depression.^74^ Thus, depressive symptoms could be a confounder or mediator of the pain–mortality relationship depending on temporal order. To assess whether treating depressive symptoms as a mediator rather than a confounder would affect our results, all analyses were repeated without adjusting for depressive symptoms in the propensity score models, regression-adjusted matching models, or doubly robust IPW models.

#### 2.3.3. Cox proportional hazard models

Cox proportional hazard models with pain exposure as a covariate were fitted to the propensity score matched samples and to the IPW samples to estimate the hazard ratio (HR) of pain exposure vs nonexposure. Cox models are a widely used approach for survival analysis^45^ that can be combined with propensity score methods in causal analyses.^9^ Pain exposure HRs and 95% confidence intervals (CIs) for the HRs are reported for all models. The estimated HRs are interpreted as the instantaneous mortality rate at any time during the follow-up for those who were exposed to pain compared with those who were not.^83^ Participants who were lost to follow-up during the study period were right censored in January of the year of the first wave in which they did not participate, eg, someone who participated in 1998 but did not return for the next wave in 2000 was censored in January 2000. We also fitted an unadjusted Cox proportional hazards model by pain in 1998 to the original imputations before propensity score methods were applied. We then repeated this analysis while adjusting for age, age squared, and sex as covariates. The results of these models were then compared with the analyses using propensity score methods.

#### 2.3.4. Kaplan–Meier curves

Kaplan–Meier curve plots are used to visualize survival over time stratified by pain exposure in the unadjusted data (n = 19,971) and the 20 imputed and one-to-one matched without replacement datasets. Mean survival probabilities were pooled across the 20 datasets for the matched data Kaplan–Meier curves. To our knowledge, there is no accepted convention for pooling variances across imputed and matched datasets for Kaplan–Meier plots, so CIs were not calculated for the matched data plot.

All analyses were carried out in R^68^ using the following packages: tidyverse (data preprocessing)^103^; mice (missing data imputation)^89^; MatchThem (PS matching and IPW of the imputed datasets)^66^; survival (survival models)^85^; survminer (Kaplen–Meier curve plots)^43^; and cobalt^31^ (balance plots). R code is available in the following GitHub repository: https://github.com/Eva-Ryan/hrs-pain-mortality.

#### 2.3.5. Sensitivity analyses

We conducted multiple sensitivity analyses to test the reliability of our findings.

(1) All models in our primary analysis were fitted without using HRS sample weights, similar to the approaches taken in previous studies of the causal effect of pain on mortality.^40,79^ As a sensitivity analysis, we re-ran all models with sample weighting (results summarized in Appendix 2, Supplementary Table 1, http://links.lww.com/PAIN/C93). Some research has found that incorporating sample weights in both the propensity score model and the outcome model is the most robust approach.^71^ Thus, we first applied the sample weights when fitting the logistic regression models used to calculate the propensity scores. Then, after propensity score matching, the sample weights were applied when fitting the Cox proportional hazards outcome models to the matched samples. For the IPW analyses, the propensity score weights were first multiplied by the sample weights to create new weights, and these composite weights were then applied when fitting the outcome models.^25^
(2) We explored the sensitivity of our confounder selection, specifically the inclusion of arthritis as a confounder, since arthritis may not confound the pain–mortality relationship. Similar to the alternative analysis in the main text where depressive symptoms were removed as a confounder, we repeated the analyses without adjusting for arthritis as a confounder (results summarized in Appendix 2, Supplementary Table 2, http://links.lww.com/PAIN/C93).
(3) As noted earlier, we explored different pain specifications to test the sensitivity of our pain exposure groupings (pain exposed = moderate or severe pain, not pain exposed = no or mild pain). The analyses were repeated using the alternative groupings severe pain exposure (severe pain) vs no severe pain exposure (no, mild, or moderate pain), any pain exposure (mild, moderate, or severe pain) vs no-pain exposure (no pain), and severe pain exposure (severe pain) vs no-pain exposure (no pain). The results are summarized in Appendix 2, Supplementary Tables 3–5, respectively (http://links.lww.com/PAIN/C93).
(4) We explored shorter follow-up lengths to test if the effect of pain exposure in 1998 on subsequent mortality is weakened over time. The analyses were repeated for 1-, 5-, and 10-year follow-up periods. The results are summarized in Appendix 2, Supplementary Table 6 (http://links.lww.com/PAIN/C93).
(5) To provide reassurance that our defined pain exposure is likely to be capturing persistent rather than transient pain, we re-ran our analyses with our exposure group restricted to “moderate/severe pain AND arthritis” with “none/mild pain” as the comparison group as in the main analysis. We reason that if a person reports both pain and arthritis, that person is likely to be referring to chronic pain (resulting from their arthritis). We suggest that if the results are similar to our original analysis, then our original exposure definition likely also refers primarily to persistent pain. The results are summarized in Appendix 2, Supplementary Table 7 (http://links.lww.com/PAIN/C93).

## 3. Results

**Figure 2.**
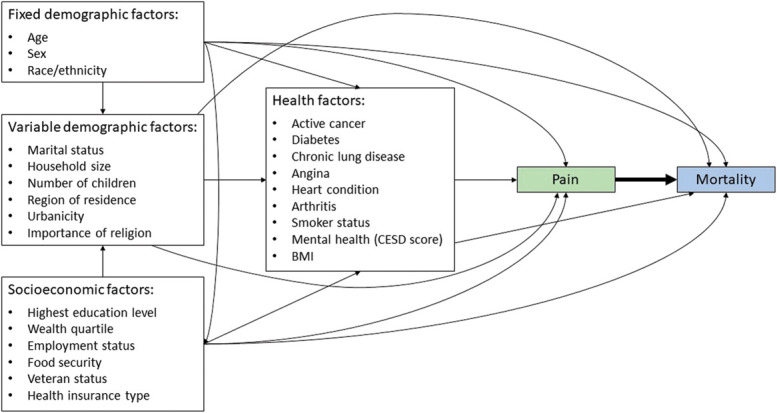
Directed acyclic graph showing assumed causal structure underlying the effect of pain (exposure) on mortality (outcome). Individual variables are grouped into “super nodes” in the DAG to improve readability. Some relationships between confounder variables have been simplified as a result (eg, while a causal arrow goes from “Socioeconomic factors” to “Variable demographic factors,” the individual demographic factor “Number of children” could causally affect the individual socioeconomic factor “Wealth quartile”). However, this simplification should not affect how well the selected confounder adjustment set accounts for confounding of the effect of pain on mortality. It is likely that a number of factors, such as functional ability or opioid analgesic use, mediate the causal effect of chronic pain on mortality. However, as we do not conduct any mediation analyses in this study, we have not included potential mediators of the pain–mortality relationship in our DAG. DAG, directed acyclic graph.

The assumed causal structure underlying our analysis is shown in the DAG in Figure 2. The exposure of interest (pain) is shown in green, the outcome of interest (mortality) is in blue, and confounders of the pain–mortality relationship are shown in white boxes. Directed arrows depict the assumed direction of causality between variables. The absence of an arrow between any 2 boxes indicates no assumed causal relationship.

Table 1 describes potential confounding variables for both the pain (n = 4073) and no-pain (n = 15,898) groups in the imputed datasets before propensity score methods are applied. Means/proportions are averaged across the 20 imputations. Covariate balance before applying propensity score methods is also summarized as averaged standardized mean differences across the imputations. There is an imbalance between the pain and no-pain groups on many background characteristics, as indicated by ASMD > 0.10 (in bold in Table 1). The largest imbalances are for arthritis (ASMD = 0.874), depressive symptoms (ASMD = 0.608), angina (ASMD = 0.287), heart condition (ASMD = 0.261), being female (ASMD = 0.235), and not being in the labour force (ASMD = 0.400), which are all more common in the pain group, and being employed (ASMD = 0.373), being in the top wealth quartile (ASMD = 0.272), and having a graduate degree (ASMD = 0.256), which are more common in the no-pain group.

**Table 1:** Baseline characteristics for individuals with and without pain averaged across the 20 imputed datasets.

Variable	Averages for imputed datasets (before propensity score methods applied) (n = 19,971)		
	Pain (exposed), n = 4073	No pain (unexposed), n = 15,898	Standardized mean differences
Age in years	67.40	67.00	0.05
Sex: female	0.66	0.54	**0.24**
Race/ethnicity			
White (non-Hispanic)	0.75	0.77	−0.05
Black (non-Hispanic)	0.14	0.14	0.02
Hispanic	0.09	0.08	0.06
Other (non-Hispanic)	0.02	0.02	−0.01
Marital status			
Married	0.62	0.66	−0.10
Separated/divorced	0.13	0.11	0.08
Widowed	0.22	0.20	0.05
Never married	0.03	0.03	0.00
Other	0.01	0.00	0.02
Household size	2.28	2.25	0.02
No. of children	3.27	3.17	0.05
Region			
Northeast	0.16	0.17	−0.02
Mid-west	0.24	0.25	−0.03
South	0.42	0.41	0.02
West	0.18	0.17	0.03
Urbanicity			
Urban	0.46	0.49	−0.06
Suburban	0.22	0.22	0.00
Ex-urban/rural	0.32	0.30	0.06
Religion			
Very important	0.68	0.64	0.09
Somewhat important	0.23	0.25	−0.06
Not too important	0.10	0.11	−0.06
Education level			
No degree	0.36	0.27	**0.20**
High school degree	0.54	0.55	−0.02
Four-year college degree	0.07	0.11	**−0.16**
Graduate degree	0.03	0.08	**−0.26**
Wealth quartile			
Q1	0.35	0.22	**0.27**
Q2	0.25	0.25	0.01
Q3	0.21	0.26	**−0.12**
Q4 (wealthiest)	0.18	0.27	**−0.22**
Employment status			
Employed	0.22	0.37	**−0.37**
Unemployed	0.02	0.02	−0.00
Retired	0.40	0.44	−0.08
Not in labour force	0.36	0.17	**0.40**
Food security: yes	0.87	0.92	**−0.17**
Veteran status: yes	0.19	0.27	**−0.21**
Health insurance			
Uninsured	0.06	0.06	0.01
Any private insurance	0.61	0.72	**−0.23**
Public insurance only	0.33	0.22	**0.23**
Active cancer: yes	0.03	0.02	0.07
Diabetes: yes	0.20	0.13	**0.16**
Lung disease: yes	0.16	0.08	**0.22**
Angina: yes	0.16	0.06	**0.29**
Stroke: yes	0.09	0.05	**0.12**
BMI category			
Underweight (BMI < 18.5)	0.03	0.02	0.07
Normal weight (18.5 ≤ BMI < 25)	0.31	0.38	**−0.14**
Overweight (25 ≤ BMI < 30)	0.35	0.40	−0.10
Obese 1 (30 ≤ BMI < 35)	0.19	0.15	0.09
Obese 2 (35 ≤ BMI < 40)	0.07	0.04	**0.13**
Obese 3 (BMI 40+)	0.04	0.01	**0.14**
Heart condition: yes	0.30	0.18	**0.26**
Arthritis: yes	0.81	0.47	**0.87**
Smoker status			
Never smoker	0.39	0.41	−0.03
Former smoker	0.42	0.43	−0.04
Current smoker	0.19	0.16	0.09
Depressive symptoms (CESD score)	2.75	1.34	**0.61**

Absolute standardized mean differences above the threshold of 0.1 (indicating imbalance between groups) are bolded. Pain (exposed) = moderate or severe pain; no pain (unexposed) = no or mild pain. N = 19,971; from the Health and Retirement Study, 1998.

BMI, body mass index.

**Figure 3.**
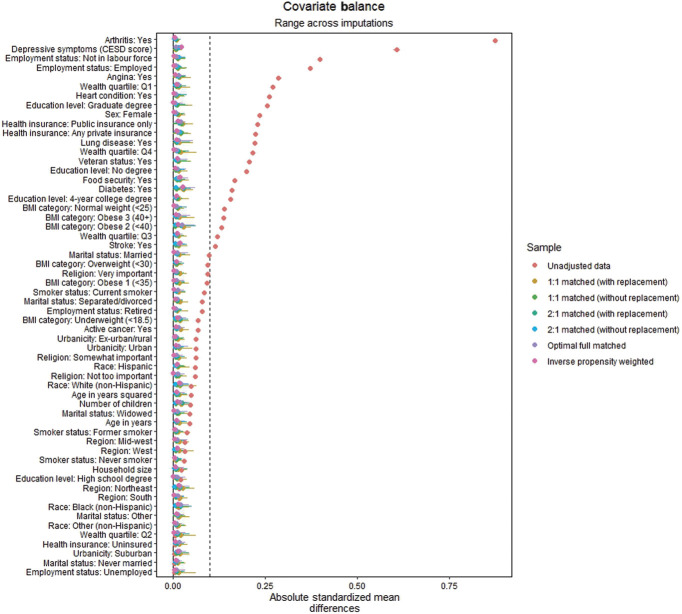
Covariate balance plot showing absolute standardized mean differences in covariate distributions between the pain (exposed) and no-pain (unexposed) groups across the 20 imputed datasets in the unadjusted data and after applying propensity score methods. The vertical dashed line at 0.1 shows the cut-off for values considered indicative of good balance. N = 19,971; from the Health and Retirement Study, 1998.

The covariate balance plot in Figure 3 compares covariate balance after applying propensity score matching or IPW with covariate balance in the unadjusted data (covariate balance summaries for the alternative matching methods are also plotted in Fig. 3 and will be discussed later). The mean and range of ASMDs for each variable across the 20 imputations are plotted as points and lines respectively. The ASMDs for the unadjusted data (plotted in red) reflect the imbalance shown in Table 1, with some large values above 0.1. Good balance is achieved across all variables by both propensity score matching without replacement (plotted in green) and propensity score weighting (plotted in pink), as all green and pink points fall below the 0.1 threshold line. Therefore, all observed confounding appears to be adjusted for by the 2 propensity score methods under consideration.

Table 2 presents the pooled Cox proportional hazard models fitted to the unadjusted imputations before any propensity score methods were applied. In the unadjusted model with only pain exposure as a covariate, the HR for those with pain vs those without was 1.32 (95% CI: 1.26-1.38). Being exposed to pain was therefore associated with a significant and substantively large increase in mortality hazard compared with being unexposed. After adjusting for age, age squared, and sex, the estimated HR for pain exposure was 1.39 (95% CI: 1.33-1.46) (second model in Table 2). Thus, the significant association between pain exposure and mortality remained after adjusting for age and sex.

**Table 2:** Pooled hazard ratio effect estimates for pain exposure in 1998 on 20-year mortality from Cox proportional hazards models fitted to the imputed samples (no propensity score methods applied).

Analysis	Hazard ratio	95% confidence interval
Unadjusted	1.32	1.26-1.38
Adjusted for age, age squared, and sex	1.39	1.33-1.46

Pain exposure = moderate or severe pain; no-pain exposure = no or mild pain. Pooled results from 20 fully imputed datasets (each n = 19,971; from the Health and Retirement Study, 1998, followed through 2018).

After fitting the propensity score models and calculating propensity scores within each imputed dataset, on average 16.5 participants in the pain group and 49.2 in the no-pain group were outside the region of common support and were discarded before propensity score matching. Matching within each imputed dataset resulted in matched samples with an average size of 6866.4 across the 20 imputed datasets, with an average of 4056.2 participants in the pain group and 2810.2 in the no-pain group. On average, less than one (0.4) participant in the pain group was unmatched and 13,038.7 participants in the no-pain group were unmatched.

**Figure 4.**
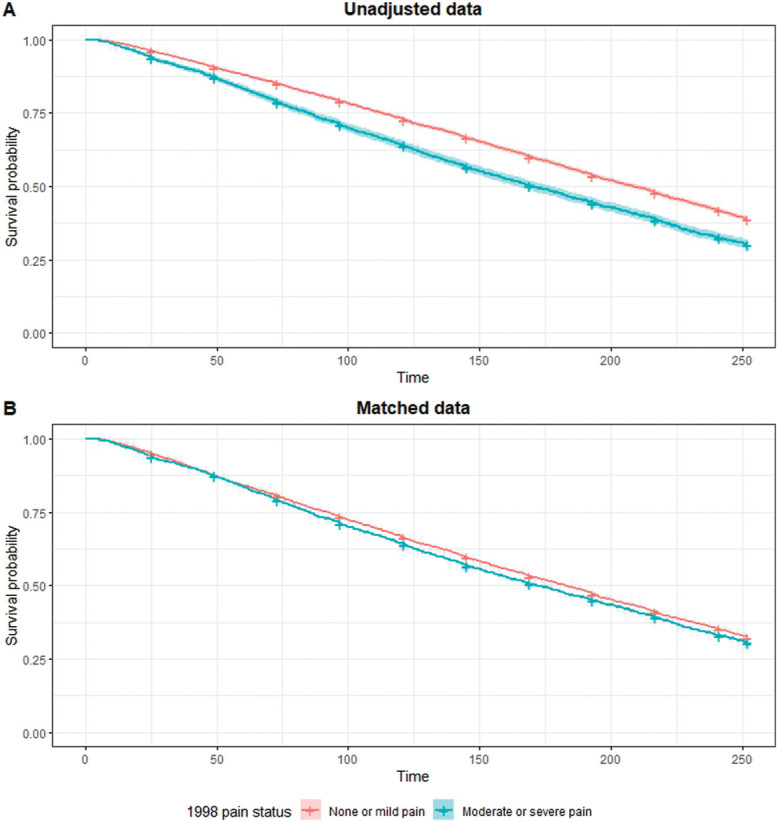
Kaplan–Meier survival curve plots stratified by pain exposure for (A) the unadjusted dataset, and (B) the 20 imputed and one-to-one propensity score matched with replacement samples. As neither pain exposure nor survival was imputed, the unadjusted data Kaplan–Meier curves were fitted using just the unadjusted dataset (n = 19,971; from the Health and Retirement Study, 1998, followed through 2018). For the one-to-one matched without replacement data Kaplan–Meier curves, mean survival probabilities were pooled across the 20 imputed and matched datasets and plotted. To our knowledge, there is no accepted convention for pooling variances across imputed and matched datasets for Kaplan–Meier plots, so confidence intervals are not calculated for this plot.

Table 3 contains the pooled results of the Cox proportional hazards models fitted to the propensity score matched samples (when using the primary one-to-one matching without replacement method). The mortality HR for those with pain was 1.06 (95% CI: 0.99-1.14). While these results are compatible with a modest positive effect of pain on mortality, they are also compatible with the possibility that pain exposure had no effect on mortality (ie, the CI includes 1.0). It is worth noting that when compared with the 32% increase in mortality hazard estimated for the pain group in the unadjusted model in Table 2, the estimated increase in mortality hazard of 6% for the pain group in the matched sample represents an 81.25% reduction in estimated effect size. Adjusting for observed confounding using propensity score matching therefore appears to account for much and potentially all of the association between pain exposure and mortality found in the comparison models in Table 2. The Kaplan–Meier curves in Figure 4 reflect this finding, with the gap between the pain exposure and no-pain exposure curves for the unadjusted data appearing wider (Fig. 4A) than the gap between the averaged pain exposure and no-pain exposure curves for the matched datasets (Fig. 4B)—visually showing that much or all of the mortality gap appears to be explained by the included confounders.

**Table 3:** Pooled hazard ratio effect estimates for pain exposure in 1998 on 20-year mortality from Cox proportional hazards models, fitted to samples created using the primary propensity score matching technique and inverse probability weighting.

Analysis	Hazard ratio	95% confidence interval
One-to-one matching without replacement	1.06	0.99-1.14
Inverse probability weighting	1.05	0.99-1.10
Regression-adjusted one-to-one matching without replacement	1.09	1.02-1.16
Doubly robust inverse probability weighted	1.06	1.00-1.12

Pain exposure = moderate or severe pain; no-pain exposure = no or mild pain. Pooled results from 20 fully imputed datasets (each n = 19,971; from the Health and Retirement Study, 1998, followed through 2018). Sample sizes (effective sample sizes) vary depending on matching (weighting) for each imputed sample.

Inverse probability weighting resulted in an average effective sample of 4073 participants in the pain group and 4796.8 in the no-pain group across the 20 imputations. The results of the pooled Cox models fitted to the IPW samples, also shown in Table 3, were similar to the propensity score matching analysis, yielding an HR of 1.05 (95% CI: 0.99-1.10). Thus, the IPW analysis is also suggestive of a modest causal effect of pain on mortality, although the 95% CI is again compatible with the possibility of no causal effect (contains 1.0).

The results of the regression-adjusted propensity score matching and the doubly robust IPW analyses are also summarized in Table 3. The HR for the regression-adjusted matching was 1.09 (95% CI = [1.02-1.16]). This analysis is more strongly suggestive of a causal effect of pain exposure on mortality, with the mortality hazard at any time over follow-up estimated to be 2% to 16% higher for those exposed to pain compared to if they had not been exposed to pain. Similarly, the doubly robust IPW method is most compatible with a modest causal effect of pain on mortality, with its 95% CI just containing 1.0 (HR = 1.06, 95% CI: 1.00-1.12).

The results from multiple alternative matching methods are provided in Table 4. Good balance was achieved by all additional matching methods, as shown in the covariate balance plot in Figure 3. In brief, almost all additional matching methods gave similar results to the one-to-one nearest neighbour matching without replacement shown in Table 3. All 95% CIs for the HR were compatible with a null or modest positive effect of pain on mortality hazard, with the exception of regression-adjusted 2-to-1 matching without replacement (HR = 1.08, 95% CI: 1.02-1.15), which resembled the results from the regression-adjusted one-to-one matching without replacement.

**Table 4:** Pooled hazard ratio effect estimates for pain exposure in 1998 on 20-year mortality from Cox proportional hazards models, fitted to matched samples created using alternative propensity score matching techniques.

Analysis	Hazard ratio	95% confidence interval
One-to-one matching with replacement	1.05	0.97-1.14
Two-to-one matching with replacement	1.05	0.97-1.13
Two-to-one matching without replacement	1.06	1.00-1.12
Optimal full matching	1.05	0.98-1.13
Regression-adjusted one-to-one matching with replacement	1.07	0.98-1.17
Regression-adjusted 2-to-1 matching with replacement	1.07	0.99-1.15
Regression-adjusted 2-to-1 matching without replacement	1.08	1.02-1.15
Regression-adjusted optimal full matching	1.06	0.97-1.14

Pain exposure = moderate or severe pain; no-pain exposure = no or mild pain. Pooled results from 20 fully imputed datasets (each n = 19,971; from the Health and Retirement Study, 1998, followed through 2018). Sample sizes vary depending on matching for each imputed sample.

Table 5 shows the results from analyses like the above except *without* adjusting for depressive symptoms. With depressive symptoms now positioned as a mediator rather than a confounder of the pain–mortality relationship, nearly all methods were most compatible with a modest positive causal effect of pain exposure on mortality, with HRs ranging from 1.06 to 1.12 and CIs nearly always excluding 1.0.

**Table 5:** Pooled hazard ratio effect estimates for pain exposure in 1998 on 20-year mortality estimated using all matching/weighting techniques, without depressive symptoms included as a covariate in the propensity score models or Cox proportional hazards models.

Analysis	Hazard ratio	95% confidence interval
One-to-one matching with replacement	1.06	0.98-1.15
One-to-one matching without replacement	1.08	1.01-1.15
Two-to-one matching with replacement	1.06	0.99-1.14
Two-to-one matching without replacement	1.08	1.02-1.14
Optimal full matching	1.06	1.00-1.13
Inverse probability weighting	1.06	1.01-1.11
Regression-adjusted one-to-one matching with replacement	1.10	1.01-1.19
Regression-adjusted one-to-one matching without replacement	1.12	1.05-1.20
Regression-adjusted 2-to-1 matching with replacement	1.10	1.02-1.18
Regression-adjusted 2-to-1 matching without replacement	1.12	1.05-1.18
Regression-adjusted optimal full matching	1.08	1.01-1.16
Doubly robust inverse probability weighted	1.09	1.03-1.15

Pain exposure = moderate or severe pain; no-pain exposure = no or mild pain. Pooled results from 20 fully imputed datasets (each n = 19,971; from the Health and Retirement Study, 1998, followed through 2018). Sample sizes (effective sample sizes) vary depending on matching (weighting) for each imputed sample.

Overall, our sensitivity analyses indicate that our main results are robust to alternate modelling decisions. Repeating the analyses with HRS sample weights gave very similar results across all matching and IPW approaches (Supplementary Table 1, http://links.lww.com/PAIN/C93). The results from analyses excluding arthritis as a confounder were also very similar to the main analyses, except that the regression-adjusted matching methods without replacement also yielded 95% CIs containing 1.0 (Supplementary Table 2, http://links.lww.com/PAIN/C93). Analyses of the effect of severe pain vs no, mild, or moderate pain (Supplementary Table 3, http://links.lww.com/PAIN/C93), mild, moderate, or severe pain vs no pain (Supplementary Table 4, http://links.lww.com/PAIN/C93), and severe pain vs no pain (Supplementary Table 5, http://links.lww.com/PAIN/C93) again gave similar results to the main analysis (with pain exposure defined as moderate or severe pain). Reducing the follow-up length to 1, 5, or 10 years resulted in wider HR CIs (Supplementary Table 6, http://links.lww.com/PAIN/C93) compared with those in Tables 2 and 3, likely due to fewer deaths occurring over follow-up. All estimated HR CIs for the 1- and 5-year follow-ups contained 1.0, providing little insight into the strength or direction of the potential causal effect. The results for the 10-year follow-up were similar to the main 20-year follow-up analysis. Models that defined exposure as having moderate/severe pain and arthritis (and thus were particularly likely to capture chronic pain) yielded results very similar to our main analysis.

## 4. Discussion

This study used propensity score methods to rigorously explore how experiencing moderate or severe pain in 1998 influenced 20-year mortality in American older adults. Although we aimed to answer whether pain causally increases mortality risk, findings were equivocal. Models consistently yielded estimated HRs slightly above 1 and were therefore compatible with pain causing a small increase in mortality hazard, even after using propensity score methods to adjust for potential confounding by a large set of sociodemographic and health-related variables. Simultaneously, many models were also compatible with no causal effect, with only a minority of CIs excluding 1. On balance, our results are likely consistent with a modest causal effect of pain on mortality. However, replicating this finding using alternative data sources will be an important task for future research.

Before applying propensity score methods, we estimated Cox proportional hazard models of mortality including only pain as a predictor or including pain and adjusting only for age, age squared, and sex. Both models showed strong associations between pain and mortality, with mortality hazards over 30% higher for individuals with pain. However, in our many propensity score matching and inverse-probability weight models, much smaller associations were found: mortality hazard was estimated to increase by 5% to 9% for individuals with pain, and many CIs were also compatible with no causal effect. For context, the causal effect we estimate is of a similar magnitude to the estimated effect of a 1-to-2-unit increase in BMI on mortality risk in the UK Biobank (HR per 1 unit increase in BMI: 1.03, 95% CI: 0.99-1.07).^98^ Models that *excluded* depressive symptoms as a potential confounder were more consistently supportive of a modest causal effect (HRs between 1.06 and 1.12), signalling the sensitivity of our findings to this particular modelling decision. Overall, this study provides some evidence that pain itself, rather than the social or medical conditions that cause it, raises mortality risk. However, our findings are consistent with much or even perhaps all of the association being driven by upstream factors that increase the risk of both pain and mortality.

While existing research on pain and mortality primarily discusses association rather than causation, a handful of studies have explicitly examined the causal nature of the relationship. Some studies have found stronger evidence than ours to suggest that pain increases mortality risk. Evidence of a causal effect was found in the ELSA (a sister study of the HRS),^79^ and a significant causal effect was found in a different American cohort including younger adults (aged ≥20 years).^40^ Our results may differ for a number of reasons, including different follow-up lengths and analytical approaches. In addition, we note that our findings may not necessarily generalize to other countries or age groups. For example, features of national healthcare systems (eg, medical costs, or percent uninsured) may shape the pain–mortality link. Previous work has also found that pain correlates differ across age groups.^105^ The question of generalizability begs for further analyses using large international cohorts.

Moreover, previous studies used different confounder adjustment sets. For example, the ELSA study used just age, sex, education, and wealth as potential confounders and posed lifestyle, health, social, and psychological factors as potential mediators. In our study, the position of each variable in the underlying causal structure was carefully considered when identifying confounders. We aimed to remove confounder bias by adjusting for all measured confounders,^36^ while avoiding the introduction of bias by adjusting for nonconfounders.^93,95^ We used a comprehensive set of potential confounders and conducted sensitivity analyses when a variable's position in the causal model was ambiguous. Our study also differs methodologically from earlier studies. Propensity score methods are preferable to traditional regression adjustments typically used in previous analyses, as they separate the study design from the outcome model by adjusting for confounding using propensity scores that are agnostic to the outcome, thus more closely emulating a randomised control trial.^2^

Our study has several implications for clinicians and public health advocates. Clinicians should be aware that chronic pain is predictive of mortality, suggesting that patients with pain should be closely monitored. Since the pain–mortality association is likely in part causal, optimally managing pain might improve not only patients' quality of life but also their quantity of life. However, since our analyses also showed that much of the association between chronic pain and mortality is attributable to confounding, upstream factors that may raise the risk of both pain and death should also be addressed. Body mass index and depressive symptoms are 2 such factors adjusted for in our analyses. There is much research linking BMI to pain^67^ and excess mortality.^28,86^ Previous work has also suggested an association between depression and pain^46^ and a dose-response effect between depressive symptoms and mortality.^73,102^ We also adjusted for several measures of low SES, which a voluminous literature has shown raises the risk of pain and mortality.^26^ These upstream factors may be more important drivers of excess mortality than pain itself and should be the subject of further investigation and public policies. Policies that reduce the risk of pain will, in many cases, also be policies to increase life expectancy.

One limitation of our study is that our pain variable, which specifies no particular duration, is not equivalent to the common definition of chronic pain as pain lasting over 3 months. We also lack information about the specific location or cause of pain. However, somewhat reassuringly, we note that the largest imbalances between our pain and no-pain groups before confounder adjustment were on characteristics known to be associated with chronic pain development, such as arthritis,^62,99^ education level, income level, and employment status.^58^ Future research may explore different datasets to better understand how findings vary across pain measures.

Our study is also limited by only considering pain at baseline. Research modelling pain exposure and confounders at multiple time points could clarify the potential cumulative causal effect of persistent pain on mortality risk. However, such modelling is complicated by exposure–confounder feedback loops, whereby historic pain is likely to causally influence confounders of pain (eg, depressive symptoms) in the future. In such cases, regression models and propensity score methods are unable to adjust for confounding without introducing other biases.^36^ Future work will require advanced causal analysis methods, such as the g-formula, to appropriately handle feedback loops.^61^

Another limitation of all causal analyses of observational data is the unverifiable assumption that the causal structure underlying the analysis is correct and all confounders of the exposure-outcome relationship are appropriately adjusted for. We conducted sensitivity analyses to investigate how altering our confounder adjustment set would change results. While removing arthritis did not significantly alter interpretations, removing depressive symptoms shifted the results to more strongly suggest a positive effect of pain on mortality. This may be because depressive symptoms confound the pain–mortality relationship, so not adjusting for them is an error that creates a spurious association. However, it is also possible that depressive symptoms are a mediator of the pain–mortality relationship, so not adjusting for them “unblocks” the causal path from pain to mortality and permits correct estimation of the causal effect. As pain and some potential confounders were reported at the same wave in our data, it was difficult to determine temporal order. This could be addressed in future work using complex time-varying analyses which allow different factors to be posed as both confounders and mediators depending on temporal order. The lack of data on some potential confounders in the 1998 HRS Wave is also a limitation. For example, some psychological factors believed to affect pain outcomes^18^ were not measured.

Conducting mediation analyses^94,97^ to investigate potential mediators of the pain–mortality relationship is another important aim for future work. Numerous mechanisms or pathways through which chronic pain may increase mortality risk can be postulated for further investigation. These may include biological pathways (eg, pain potentially causing cardiovascular damage^27^), pain management strategies linked to increased mortality risk (eg, opioid analgesic use^40^), or pain having a negative effect on overall health and wellbeing (eg, by limiting physical activity^15^).

To our knowledge, this is the first study to use propensity score matching and IPW to investigate the potentially causal association between pain and mortality. We aimed to conduct a precise and comprehensive analysis of this relationship, adjusting for many reasonable confounders and applying various supplementary analyses to test the results' robustness. Our findings were mixed, comprising some analyses that clearly indicated a modest causal effect of pain on mortality, but also a greater number of analyses pointing in the same direction but compatible with no causal effect. This topic warrants further investigation with alternate data sources and modelling strategies. Nonetheless, the substantial attenuation of the observed association after confounder adjustment highlights the large role of potentially modifiable upstream risk factors for both pain and mortality. This work provides a basis for future studies examining the potential causal effect of pain on mortality in different countries and contexts, using different measures of pain, exploring potential mediators of the pain–mortality causal relationship, and considering the potential cumulative causal effect of pain at multiple time points.

## Conflict of interest statement

The authors have no conflicts of interest to declare.

## Supplementary Material

SUPPLEMENTARY MATERIAL

## Supplemental digital content

Supplemental digital content associated with this article can be found online at http://links.lww.com/PAIN/C93.
