# Supplementary material for: Is the relationship between chronic pain and mortality causal? A propensity score analysis
Source: Pain. 2024 Jul 9;166(1):183–95. doi: 10.1097/j.pain.0000000000003336 (PMC11647826; doi:10.1097/j.pain.0000000000003336)
Supplement: SUPPLEMENTARY MATERIAL [file jop-166-183-s001.pdf]

APPENDIX 1

Propensity score matching

Propensity score matching involves pairing participants in the exposed group with participants in the unexposed group such that paired participants have close or identical propensity scores. This creates a matched sample of participants with similar propensities for pain, but with only one participant in a matched pair exposed to pain. One-to-one nearest neighbour caliper matching without replacement was used. This matching algorithm iterates through each pain-exposed participant and matches them to the unexposed participant with the most similar propensity score within a specified caliper distance. If there are no unexposed participants within the allowed caliper distance from the exposed participant's propensity score, the exposed participant remains unmatched. We applied a conservative caliper of 0.1 times the standard deviation of the propensity scores. When matching without replacement, once an unexposed participant is matched to an exposed participant they cannot be matched with another exposed participant. Nearest neighbour caliper matching without replacement has been found to outperform other matching algorithms [1] including matching with replacement, which allows unexposed participants to be matched to multiple exposed participants. The region of common support refers to the range of propensity score values for which the exposed and unexposed groups overlap. A large overlap is required for successful matching. If a small number of scores fall outside this region it is recommended to discard them [5; 6].

Alternative propensity score matching methods

The first alternative propensity score matching method applied was matching with replacement rather than without, meaning one unexposed participant could be matched to multiple exposed participants. We also applied two-to-one matching, meaning two unexposed participants were matched to each exposed participant. Both of these alterations can increase the number of matches made thus increasing the matched sample size. Optimal full matching was also conducted which involves subdividing the sample into a number of matched sets such that each set contains one exposed participant and ≥ 1 unexposed participant, or one unexposed participant and ≥ 1 exposed participant. The algorithm is considered optimal as the total distance in propensity scores between members of the same subsets is minimized [8].

Inverse probability weighting

Inverse probability weighting (IPW) involves creating weights from the calculated propensity scores such that, after applying the weights, the exposed and unexposed groups have similar background characteristic distributions and differ only by exposure. As was also the case for propensity score

matching, the causal effect we wish to estimate using IPW is the average treatment effect in the treated (ATT), meaning the average effect of pain exposure on mortality for those who were actually exposed to pain [2]. As those who were exposed to pain are the target population, these participants are assigned a weight of one. To weigh the unexposed group such that they have a distribution of background characteristics similar to the exposed group, those who were not exposed to pain are assigned a weight using the formula

$$w = \frac{P(Z=1|\boldsymbol{X})}{1- P(Z=1\ |\boldsymbol{X})},$$

where $P(Z = 1|\boldsymbol{X})$ is the propensity score, the probability of exposure to pain ($Z = 1$) given the vector of observed background characteristics $\boldsymbol{X}$. If an unexposed participant has a very high propensity score this can result in a very large weight, which can increase the variability of the causal effect estimate. We thus stabilized the weights by multiplying each participant's weight by the proportion of participants in their exposure group [4; 9].

Doubly robust models and regression-adjusted matching

While the accuracy of effect estimates obtained using propensity score matching or IPW alone rely on correct specification of the propensity score model for the exposure, doubly robust methods and regression-adjusted matching also adjust for confounding in the outcome model to safeguard against misspecification of the propensity score model [10]. In brief, doubly robust methods involve first fitting a propensity score model for the exposure to estimate inverse probability weights, then after applying the weights a second model is fitted for the outcome (in this case, a Cox regression model) with covariate adjustment for both confounders and exposure status. Only one of the two models need to be specified correctly for the effect estimate to be unbiased [7]. Similarly, regression-adjusted matching involves first creating a propensity score matched sample as described above, then fitting an outcome model with covariate adjustment for confounders and exposure status [3].

APPENDIX 2

Supplementary Table 1: Pooled hazard ratio effect estimates for pain exposure in 1998 on 20-year mortality estimated using all matching/weighting techniques, with HRS sample weights.

Analysis	Hazard Ratio	95% Confidence Interval
One-to-one matching with replacement	1.07	(0.97, 1.18)
One-to-one matching without replacement	1.08	(1.00, 1.16)
Two-to-one matching with replacement	1.06	(0.98, 1.15)
Two-to-one matching without replacement	1.07	(1.00, 1.14)
Optimal full matching	1.06	(0.97, 1.15)
Inverse probability weighting	1.06	(1.00, 1.11)
Regression-adjusted one-to-one matching with replacement	1.10	(0.99, 1.22)
Regression-adjusted one-to-one matching without replacement	1.10	(1.02, 1.19)
Regression-adjusted two-to-one matching with replacement	1.09	(1.00, 1.18)
Regression-adjusted two-to-one matching without replacement	1.09	(1.02, 1.16)
Regression-adjusted optimal full matching	1.07	(0.97, 1.17)
Doubly robust inverse probability weighted	1.06	(0.99, 1.12)

Note: Pain exposure = moderate or severe pain; no pain exposure = no or mild pain. Pooled results from 20 fully imputed datasets (each n = 19,971; from the Health and Retirement Study, 1998, followed through 2018). Sample sizes (effective sample sizes) vary depending on matching (weighting) for each imputed sample.

Supplementary Table 2: Pooled hazard ratio effect estimates for pain exposure in 1998 on 20-year mortality estimated using all matching/weighting techniques, without arthritis included as a covariate in the propensity score models or Cox proportional hazards models.

Analysis	Hazard Ratio	95% Confidence Interval
One-to-one matching with replacement	1.03	(0.95, 1.11)
One-to-one matching without replacement	1.04	(0.97, 1.11)
Two-to-one matching with replacement	1.03	(0.96, 1.09)
Two-to-one matching without replacement	1.04	(0.99, 1.10)
Optimal full matching	1.03	(0.96, 1.10)
Inverse probability weighting	1.03	(0.98, 1.08)
Regression-adjusted one-to-one matching with replacement	1.05	(0.96, 1.14)
Regression-adjusted one-to-one matching without replacement	1.07	(1.00, 1.14)
Regression-adjusted two-to-one matching with replacement	1.04	(0.97, 1.12)
Regression-adjusted two-to-one matching without replacement	1.06	(1.00, 1.12)
Regression-adjusted optimal full matching	1.03	(0.96, 1.12)
Doubly robust inverse probability weighted	1.04	(0.98, 1.10)

Note: Pain exposure = moderate or severe pain; no pain exposure = no or mild pain. Pooled results from 20 fully imputed datasets (each n = 19,971; from the Health and Retirement Study, 1998, followed through 2018). Sample sizes (effective sample sizes) vary depending on matching (weighting) for each imputed sample.

Supplementary Table 3: Pooled hazard ratio effect estimates for severe pain exposure versus no severe pain exposure in 1998 on 20-year mortality estimated using all matching/weighting techniques considered.

Analysis	Hazard Ratio	95% Confidence Interval
One-to-one matching with replacement	1.07	(0.93, 1.22)
One-to-one matching without replacement	1.07	(0.94, 1.21)
Two-to-one matching with replacement	1.07	(0.96, 1.19)
Two-to-one matching without replacement	1.07	(0.97, 1.18)
Optimal full matching	1.07	(0.97, 1.18)
Inverse probability weighting	1.09	(1.00, 1.18)
Regression-adjusted one-to-one matching with replacement	1.13	(0.97, 1.30)
Regression-adjusted one-to-one matching without replacement	1.12	(0.98, 1.27)
Regression-adjusted two-to-one matching with replacement	1.12	(1.00, 1.26)
Regression-adjusted two-to-one matching without replacement	1.12	(1.01, 1.25)
Regression-adjusted optimal full matching	1.09	(0.99, 1.21)
Doubly robust inverse probability weighted	1.12	(1.03, 1.22)

Note: Severe pain exposure = severe pain; no severe pain exposure = no, mild, or moderate pain. Pooled results from 20 fully imputed datasets (each n = 19,971; from the Health and Retirement Study, 1998, followed through 2018). Sample sizes (effective sample sizes) vary depending on matching (weighting) for each imputed sample.

Supplementary Table 4: Pooled hazard ratio effect estimates for <u>any</u> pain exposure versus <u>no</u> pain exposure in 1998 on 20-year mortality estimated using all matching/weighting techniques considered.

Analysis	Hazard Ratio	95% Confidence Interval
One-to-one matching with replacement	1.05	(0.97, 1.14)
One-to-one matching without replacement	1.07	(1.02, 1.14)
Two-to-one matching with replacement	1.05	(0.99, 1.12)
Two-to-one matching without replacement	1.08	(1.02, 1.13)
Optimal full matching	1.05	(0.98, 1.12)
Inverse probability weighting	1.04	(0.99, 1.10)
Regression-adjusted one-to-one matching with replacement	1.08	(0.99, 1.16)
Regression-adjusted one-to-one matching without replacement	1.09	(1.03, 1.16)
Regression-adjusted two-to-one matching with replacement	1.07	(1.00, 1.15)
Regression-adjusted two-to-one matching without replacement	1.09	(1.03, 1.15)
Regression-adjusted optimal full matching	1.06	(0.99, 1.13)
Doubly robust inverse probability weighted	1.07	(1.01, 1.13)

Note: Any pain exposure = mild, moderate or severe pain; no pain exposure = no pain. Pooled results from 20 fully imputed datasets (each n = 19,971; from the Health and Retirement Study, 1998, followed through 2018). Sample sizes (effective sample sizes) vary depending on matching (weighting) for each imputed sample.

Supplementary Table 5: Pooled hazard ratio effect estimates for severe pain exposure versus no pain exposure in 1998 on 20-year mortality estimated using all matching/weighting techniques considered.

Analysis	Hazard Ratio	95% Confidence Interval
One-to-one matching with replacement	1.06	(0.91, 1.24)
One-to-one matching without replacement	1.07	(0.93, 1.23)
Two-to-one matching with replacement	1.06	(0.94, 1.20)
Two-to-one matching without replacement	1.07	(0.96, 1.19)
Optimal full matching	1.06	(0.94, 1.20)
Inverse probability weighting	1.06	(0.97, 1.17)
Regression-adjusted one-to-one matching with replacement	1.12	(0.96, 1.32)
Regression-adjusted one-to-one matching without replacement	1.14	(1.00, 1.30)
Regression-adjusted two-to-one matching with replacement	1.11	(0.98, 1.27)
Regression-adjusted two-to-one matching without replacement	1.13	(1.01, 1.26)
Regression-adjusted optimal full matching	1.08	(0.94, 1.23)
Doubly robust inverse probability weighted	1.09	(0.98, 1.21)

Note: Severe pain exposure = severe pain; no pain exposure = no pain. Pooled results from 20 fully imputed datasets (each n = 19,971; from the Health and Retirement Study, 1998, followed through 2018). Sample sizes (effective sample sizes) vary depending on matching (weighting) for each imputed sample.

Supplementary Table 6: Pooled hazard ratio effect estimates (confidence intervals in brackets) for pain exposure in 1998 on mortality over 1, 5, and 10-year follow-ups, estimated using all matching/weighting techniques considered.

Analysis	1 year follow-up	5 year follow-up	10 year follow-up
One-to-one matching with replacement	1.17 (0.69, 1.99)	1.04 (0.89, 1.21)	1.08 (0.97, 1.20)
One-to-one matching without replacement	1.16 (0.78, 1.73)	1.03 (0.91, 1.17)	1.08 (0.99, 1.19)
Two-to-one matching with replacement	1.16 (0.74, 1.80)	1.03 (0.90, 1.18)	1.07 (0.97, 1.18)
Two-to-one matching without replacement	1.16 (0.82, 1.64)	1.02 (0.91, 1.14)	1.08 (1.00, 1.16)
Optimal full matching	1.15 (0.76, 1.73)	1.03 (0.91, 1.18)	1.07 (0.98, 1.18)
Inverse probability weighting	1.14 (0.81, 1.59)	1.03 (0.93, 1.15)	1.06 (0.99, 1.15)
Regression-adjusted one-to-one matching with replacement	1.14 (0.66, 1.99)	1.07 (0.90, 1.27)	1.11 (0.98, 1.26)
Regression-adjusted one-to-one matching without replacement	1.15 (0.76, 1.75)	1.07 (0.94, 1.22)	1.12 (1.03, 1.23)
Regression-adjusted two-to-one matching with replacement	1.14 (0.71, 1.83)	1.06 (0.92, 1.23)	1.10 (1.00, 1.22)
Regression-adjusted two-to-one matching without replacement	1.15 (0.80, 1.66)	1.05 (0.94, 1.18)	1.11 (1.03, 1.21)
Regression-adjusted optimal full matching	1.11 (0.71, 1.73)	1.05 (0.91, 1.21)	1.09 (0.98, 1.21)
Doubly robust inverse probability weighted	1.12 (0.79, 1.59)	1.06 (0.95, 1.19)	1.09 (1.01, 1.18)

Note: Pain exposure = moderate or severe pain; no pain exposure = no or mild pain. Pooled results from 20 fully imputed datasets (each n = 19,971; from the Health and Retirement Study, 1998, followed through 2018). Sample sizes (effective sample sizes) vary depending on matching (weighting) for each imputed sample.

Supplementary Table 7: Pooled hazard ratio effect estimates for pain AND arthritis exposure versus no pain exposure in 1998 on 20-year mortality estimated using all matching/weighting techniques considered.

Analysis	Hazard Ratio	95% Confidence Interval
One-to-one matching with replacement	1.02	(0.93, 1.12)
One-to-one matching without replacement	1.04	(0.96, 1.12)
Two-to-one matching with replacement	1.02	(0.94, 1.10)
Two-to-one matching without replacement	1.04	(0.97, 1.10)
Optimal full matching	1.02	(0.94, 1.10)
Inverse probability weighting	1.02	(0.96, 1.09)
Regression-adjusted one-to-one matching with replacement	1.05	(0.96, 1.15)
Regression-adjusted one-to-one matching without replacement	1.07	(0.99, 1.15)
Regression-adjusted two-to-one matching with replacement	1.05	(0.97, 1.14)
Regression-adjusted two-to-one matching without replacement	1.06	(1.00, 1.14)
Regression-adjusted optimal full matching	1.04	(0.96, 1.13)
Doubly robust inverse probability weighted	1.04	(0.97, 1.11)

Note: Pain AND arthritis exposure = severe or moderate pain AND arthritis; no pain exposure = no or mild pain (with or without arthritis). Pooled results from 20 fully imputed datasets (each n = 19,971; from the Health and Retirement Study, 1998, followed through 2018). Sample sizes (effective sample sizes) vary depending on matching (weighting) for each imputed sample.

REFERENCES

[1] Austin PC. A comparison of 12 algorithms for matching on the propensity score. Statistics in Medicine 2014;33(6):1057-1069.
[2] Austin PC. The use of propensity score methods with survival or time-to-event outcomes: reporting measures of effect similar to those used in randomized experiments. Statistics in Medicine 2014;33(7):1242-1258.
[3] Austin PC. Double propensity-score adjustment: a solution to design bias or bias due to incomplete matching. Statistical Methods in Medical Research 2017;26(1):201-222.
[4] Austin PC, Stuart EA. Moving towards best practice when using inverse probability of treatment weighting (IPTW) using the propensity score to estimate causal treatment effects in observational studies. Statistics in Medicine 2015;34(28):3661-3679.
[5] Caliendo M, Kopeinig S. Some practical guidance for the implementation of propensity score matching. Journal of Economic Surveys 2008;22(1):31-72.
[6] Dennison CR. The crime-reducing benefits of a college degree: evidence from a nationally representative US sample. Criminal Justice Studies 2019;32(4):297-316.
[7] Funk MJ, Westreich D, Wiesen C, Stürmer T, Brookhart MA, Davidian M. Doubly robust estimation of causal effects. American Journal of Epidemiology 2011;173(7):761-767.
[8] Hansen BB. Full matching in an observational study of coaching for the SAT. Journal of the American Statistical Association 2004;99(467):609-618.
[9] Hernán MA, Robins JM. Causal Inference: What If: Boca Raton: Chapman & Hall/CRC, 2020.
[10] Kreif N, Grieve R, Radice R, Sekhon JS. Regression-adjusted matching and double-robust methods for estimating average treatment effects in health economic evaluation. Health Services and Outcomes Research Methodology 2013;13:174-202.
